# Tumor Volume Index as a Predictor of Pelvic Lymph Node Metastasis in Low-Risk Endometrial Cancer

**DOI:** 10.7759/cureus.79836

**Published:** 2025-02-28

**Authors:** Yuji Tanaka, Tsukuru Amano, Akimasa Takahashi, Yutaka Yoneoka, Ayako Inatomi, Mari Deguchi, Hiroyuki Yamanaka, Yuri Nobuta, Shunichiro Tsuji, Takashi Murakami

**Affiliations:** 1 Department of Obstetrics and Gynecology, Shiga University of Medical Science, Otsu, JPN

**Keywords:** endometrial cancer, endometrioid carcinoma, low-risk endometrial cancer, lymph node metastasis, mri, prognostic factor, risk assessment, risk stratification, tumor volume, tumor volume index

## Abstract

This study aimed to identify predictors of pelvic lymph node metastasis in low-risk endometrial cancer, defined as cases with no more than half myometrial invasion, preoperative endometrial biopsy results indicating endometrioid carcinoma Grade 1 (G1) or Grade 2 (G2), and no extrauterine spread. Among the factors examined, we focused on the tumor volume index derived from MRI, calculated by multiplying the maximum longitudinal diameter along the uterine axis, the maximum anteroposterior diameter on the sagittal plane, and the maximum transverse diameter on the horizontal plane. A retrospective analysis was conducted on 117 patients who underwent the standard treatment protocol (total hysterectomy, bilateral salpingo-oophorectomy, and pelvic lymph node dissection) at our institution from July 1, 2014, to December 31, 2023. Pelvic lymph node metastasis was observed in seven cases (5.9%). Univariate analysis showed a significant association with serum cancer antigen-125 (CA-125) level (p=0.035) and tumor volume index (p=0.003). A receiver operating characteristic (ROC) analysis revealed that a tumor volume index cutoff of 38 cm³ yielded an area under the curve (AUC) of 0.83, with a true positive fraction (TPF) of 0.86 and a false positive fraction (FPF) of 0.15. Multivariate analysis also identified a tumor volume index (≥38 cm³) as an independent predictive factor (odds ratio 26.3, 95% confidence interval 2.6-272, p=0.006). Cases with a tumor volume index ≥38 cm³ accounted for 23 cases (20% of all) of the cohort; among these, six cases (25%) had pelvic lymph node metastases. In contrast, the metastasis rate was only one case (1%) in 94 cases (80% of all) with a tumor volume index <38 cm³. These findings suggest that the tumor volume index is useful for evaluating the risk of pelvic lymph node metastasis in low-risk endometrial cancer, contributing to decision-making on whether to perform pelvic lymph node dissection and risk stratification for sentinel lymph node navigation surgery.

## Introduction

When endometrial cancer is diagnosed preoperatively with no more than half myometrial invasion and preoperative endometrial biopsy results indicating endometrioid carcinoma Grade 1 (G1) or Grade 2 (G2) without any extrauterine spread, it is defined as low-risk endometrial cancer. In such cases, the incidence of lymph node metastasis is known to be low. In the Gynecologic Oncology Group (GOG) 33 study, the incidence of lymph node metastasis was reported to be 4% for endometrioid G1 or G2 cases with less than two-thirds myometrial invasion [1]. A randomized controlled trial, A Study in the Treatment of Endometrial Cancer (ASTEC) trial (published in 2009), examined the significance of lymph node dissection in endometrial cancer confined to the uterus and concluded, through subgroup analysis, that there was no benefit to lymph node dissection in cases of G1 or G2 endometrioid carcinoma with ≤1/2 myometrial invasion [2]. A 2017 update of the Cochrane review also indicated that systematic lymph node dissection offers limited therapeutic benefit for early-stage endometrial cancer [3].

However, even among low-risk endometrial cancer cases, a subgroup with a relatively higher rate of lymph node metastasis may exist. For such cases, lymph node dissection could still have potential therapeutic value. Identifying patients within the low-risk group who are at a comparatively higher risk for lymph node metastasis is crucial to determining which cases might benefit from pelvic lymph node dissection.

In addition, if sentinel lymph node navigation surgery, which is now increasingly adopted internationally for endometrial cancer, is indicated for low-risk endometrial cancer, a preoperative evaluation of the risk of lymph node metastasis would also be beneficial.

One of the most influential studies in this domain was conducted at the Mayo Clinic, culminating in the “Algorithm for the surgical treatment of endometrial cancer” published in 2000. Based on large-scale data from the 1980s and 1990s, it advocated omitting lymph node dissection in cases of G1 or G2 endometrioid carcinoma with ≤1/2 myometrial invasion and a tumor size of ≤2 cm, as these patients have a lower rate of lymph node metastasis compared to those with tumor size >2 cm [4]. However, considering improvements in MRI diagnostic accuracy since the 1980s-1990s, revisions of the International Federation of Gynecology and Obstetrics (FIGO) staging system (in 1988, 2009, and 2023), and the increasing incidence of endometrial cancer, re-evaluation of this approach seems warranted. Note that this study uses the FIGO 2009 staging system.

At our institution, sentinel lymph node navigation surgery has been performed for low-risk endometrial cancer as of 2025. However, until 2023, pelvic lymph node dissection was routinely performed as the standard treatment for all low-risk cases, allowing us to accumulate relevant data on pelvic lymph node metastasis. Therefore, based on a retrospective analysis of cases at our institution, we aimed to identify risk factors for a relatively higher incidence of lymph node metastasis even among cases initially presumed low-risk based on preoperative assessment.

## Materials and methods

This single-institution retrospective study included patients diagnosed with endometrial cancer between July 1, 2014, and December 31, 2023, who underwent standard treatment at Shiga University of Medical Science. Specifically, low-risk endometrial cancer was defined as cases that, based on preoperative diagnosis, exhibited no more than half myometrial invasion, endometrioid carcinoma G1 or G2, and no extrauterine spread. All patients underwent total hysterectomy, bilateral salpingo-oophorectomy, and pelvic lymph node dissection (standard treatment protocol). The common iliac, external iliac, internal iliac, obturator, circumflex iliac, parametrial, and sacral lymph nodes in the pelvic area were dissected. Exclusion criteria were cases in which pelvic lymph node dissection was omitted, overlapping cancers, or cases in which accurate evaluation of tumor volume was difficult due to benign uterine conditions such as large leiomyomas. This study was approved by our institutional ethics committee (R2022-042).

**Figure 1:**
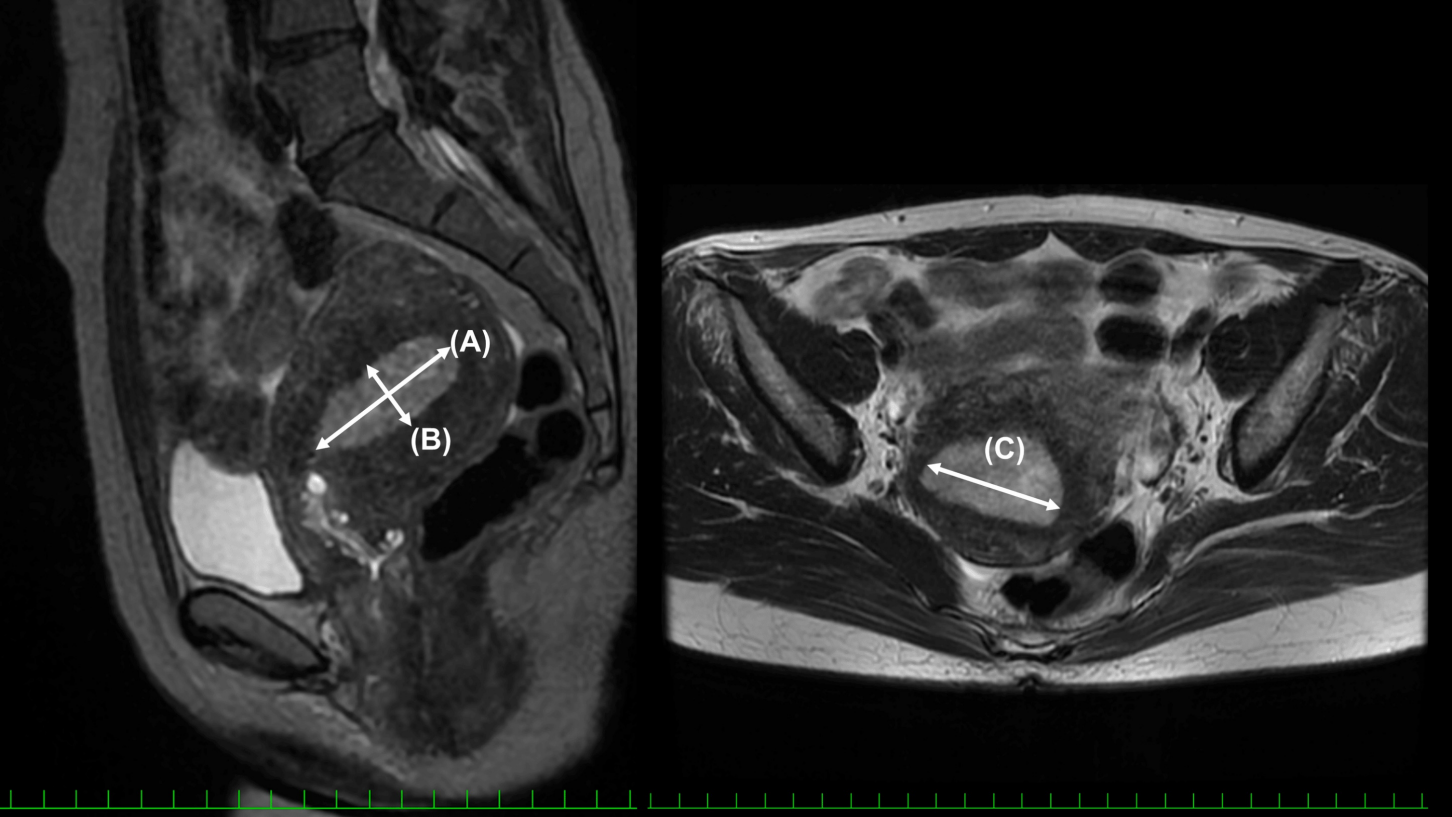
Tumor volume index. The tumor volume index is defined as the product of three measurements on T2-weighted MRI. A: the maximum longitudinal diameter along the uterine axis; B: the maximum anteroposterior diameter on the sagittal plane; and C: the maximum transverse diameter on the horizontal plane. The representative images (original to this article) demonstrate the method used to calculate the tumor volume index as described in [5].

We retrospectively collected data from medical records, including basic patient information, preoperative imaging, and perioperative and postoperative pathology reports. In analyzing tumor volume, we used the tumor volume index derived from preoperative MRI data, following previously reported methods [5]. This article is available under the terms of the Creative Commons Attribution-NonCommercial-ShareAlike License [5]. The tumor volume index was defined as the product (in cm³) of three measurements of tumor size: the maximum longitudinal diameter (in cm) along the uterine axis on T2-weighted sagittal images, the maximum anteroposterior diameter (in cm) on the sagittal plane, and the maximum transverse diameter (in cm) on the horizontal plane (Figure 1).

The staging was performed according to FIGO 2009. Continuous variables are presented as medians and interquartile ranges (IQR). Statistical analyses included chi-square tests, Mann-Whitney U tests for univariate analyses, and logistic regression for multivariate analyses. A p-value <0.05 was considered statistically significant. All data analyses were conducted using BellCurve for Excel (Tokyo, Japan).

## Results

During the study period, 153 cases were initially identified as low-risk endometrial cancer. However, 36 cases were excluded due to the omission of pelvic lymph node dissection (e.g., in elderly patients or those with significant comorbidities), overlapping cancers, or the presence of large uterine fibroids that precluded accurate tumor volume assessment. Consequently, 117 cases were analyzed, all of which underwent laparoscopic or robot-assisted surgery. Patient characteristics and perioperative outcomes are shown in Table 1. No uterine manipulator was used. The median number of dissected lymph nodes was 28.

**Table 1:** Patient characteristics and perioperative outcomes. Data are shown as median (interquartile range) or number (%); CA-125: cancer antigen-125

Parameter	Result
Age (years)	54 (49-61)
BMI (kg/m^2^)	23.8 (21-27.9)
Serum CA-125 level (U/mL)	13 (8.8-20)
Tumor volume index (cm^3^)	8.9 (2.1-26.4)
Procedure	Abdominal: 0 (0%)
	Laparoscopic: 67 (57.2%)
	Robot-assisted: 50 (42.7%)
Use of a uterine manipulator	0 (0%)
Lymph node count (number)	28 (20-40)

Postoperative outcomes are shown in Table 2. Pelvic lymph node metastasis was observed in seven patients (5.9%). Postoperative pathological diagnosis resulted in 31 patients (26.5%) being upstaged. The median follow-up period was 40 months, and the recurrence rate was three patients (2.6%).

**Table 2:** Postoperative outcomes. Data are shown as number (%), † Classified according to FIGO 2009, § Median follow-up period: 40 months FIGO: International Federation of Gynecology and Obstetrics

Parameter	Result
Stage^†^	IA: 86 (73.5%)
	IB: 9 (7.7%)
	II: 12 (10.2%)
	IIIA: 3 (2.6%)
	IIIC: 7 (6.0%)
Histologic subtype^†^	Endometrioid carcinoma G1: 90 (76.9%)
	Endometrioid carcinoma G2: 19 (16.2%)
	Endometrioid carcinoma G3: 3 (2.6%)
	Serous carcinoma: 4 (3.4%)
	Other: 1 (0.8%)
Positive pelvic lymph node metastasis	7 (5.9%)
Adnexal metastasis	5 (4.3%)
Cervical stromal invasion	17 (14.5%)
Deep myometrial invasion (≥50%)	18 (15.4%)
Positive peritoneal cytology	3 (2.6%)
Recurrence^§^	3 (2.6%)

Univariate analysis of factors associated with pelvic lymph node metastasis showed significant differences for cancer antigen-125 (CA-125) (p=0.035) and tumor volume index (p=0.003), as summarized in Table 3.

**Table 3:** Univariate analysis of factors for lymph node metastasis. Data are shown as median (interquartile range) or number (%); CA-125: cancer antigen-125

Parameter	Negative (n=110)	Metastasis (n=7)	P-value
Age (years)	54 (49-61)	55 (52-66.5)	0.47
BMI (kg/m^2^)	24 (21-29)	21 (20.9-22.4)	0.10
Serum CA-125 level (U/mL)	12 (8-19)	66 (12-103.5)	0.035
Tumor volume index (cm^3^)	8.3 (2-23)	43 (38.4-47.5)	0.003
Preoperative endometrial biopsy: endometrioid carcinoma grade	G1: 96 (87.3%)	G1: 6 (85.8%)	1.00
	G2: 14 (12.7%)	G2: 1 (14.2%)	

**Figure 2:**
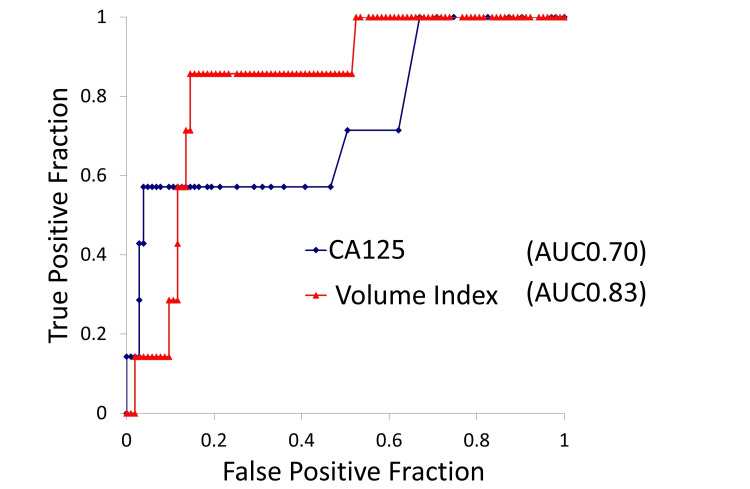
ROC analysis of potential predictors for pelvic lymph node metastasis. AUC: area under the curve; ROC: receiver operating characteristic; CA-125: cancer antigen-125

Using receiver operating characteristic (ROC) curves for the outcome of pelvic lymph node metastasis and the predictors CA-125 and tumor volume index, the area under the curve (AUC) for CA-125 was 0.70, making it less suitable for setting a clear cutoff. In contrast, a tumor volume index cutoff of 38 cm³ yielded an AUC of 0.83 with a false positive fraction (FPF) of 0.15 and a true positive fraction (TPF) of 0.86 (Figure 2).

For multivariate analysis, we used the following cutoffs: tumor volume index ≥38 cm³ (based on this study), age ≥50 years, BMI ≥23.8 (median in this study), and CA-125 ≥35 U/mL (based on previous reports) [6]. The odds ratio (95% confidence interval, p-value) was 3.7 (0.5-26.5, p=0.19) for CA-125 and 26.3 (2.6-272, p=0.006) for the tumor volume index (≥38 cm³; Table 4).

**Table 4:** Multivariate analysis of factors for lymph node metastasis. CA-125: cancer antigen-125

Parameter	Odds ratio (95% confidence interval)	P-value
Age (>50 years)	1.8 (0.15-23.3)	0.64
BMI (>23.8)	0.2 (0.03-1.66)	0.14
CA-125 (≥35 U/mL)	3.7 (0.5-26.5)	0.19
Tumor volume index (≥38 cm^3^)	26.3 (2.6-272)	0.006

Cases with a tumor volume index ≥38 cm³ accounted for 23 cases (20% of all) of the cohort; among these, six cases (25%) had pelvic lymph node metastases. In contrast, the metastasis rate was only one case (1%) in 94 cases (80% of all) with a tumor volume index <38 cm³.

## Discussion

Among cases with no more than half myometrial invasion, preoperative endometrial biopsy results indicating endometrioid carcinoma G1 or G2, and no extrauterine spread, defined as low-risk endometrial cancer, this retrospective study identified the tumor volume index as a risk factor for pelvic lymph node metastasis.

Previous studies investigating the significance of the tumor volume index in endometrial cancer have reported cutoff values of 35-36 cm³ that correlate with lymph node metastasis and prognosis, regardless of histologic subtype or stage [7,8]. Some investigations have also demonstrated the prognostic value of the tumor volume index, specifically in low-risk endometrial cancer [5]. In a study focusing exclusively on G1 endometrioid carcinoma (irrespective of stage), the combination of CA-125 ≥30 U/mL and a tumor volume index ≥36 cm³ was identified as a risk factor for lymph node metastasis [9]. Interestingly, for detecting pelvic lymph node metastasis in low-risk endometrial cancer, our data also determined a tumor volume index cutoff of approximately 38 cm³, which closely aligns with previously reported cutoff values in various contexts beyond pelvic lymph node metastasis in low-risk endometrial cancer for lymph node metastasis and prognostic factors as outcome measures.

Other predictors for lymph node metastasis have been proposed, such as a tumor diameter of 2 cm in low-risk cases [4]. Broader studies not limited to low-risk disease have suggested combined scoring systems incorporating tumor volume, depth of myometrial invasion, and CA-125 [10,11], as well as the usefulness of Ki-67 [12]. Systematic reviews and meta-analyses also often highlight the value of CA-125 and tumor volume in predicting lymph node metastasis [13-18].

From this literature review, it is evident that both tumor volume (diameter, tumor volume index, thickness, etc.) and CA-125 levels are broadly useful for detecting lymph node metastasis across various stages and histologic subtypes of endometrial cancer. Our current research focused on low-risk endometrial cancer and found the tumor volume index to be a useful predictor of lymph node metastasis. This result could be of practical significance when deciding whether pelvic lymph node dissection is necessary or when performing sentinel lymph node navigation surgery, as it helps estimate the preoperative risk of lymph node metastasis. Notably, the tumor volume index does not require specialized imaging techniques such as reduced field-of-view diffusion-weighted (rFOV) sequences, which adds to its ease of use in clinical practice [19].

Although CA-125 showed significance in univariate analysis, it did not remain significant in multivariate analysis. One possibility is that CA-125 may have lower utility, specifically in low-risk endometrial cancer. Another possibility is that approaches adjusting the cutoff according to menopausal status or age might enhance its predictive power [10]. Larger-scale investigations exploring such tailored cutoffs are warranted.

A strength of this study is that, during the study period, our institution performed pelvic lymph node dissection for all low-risk endometrial cancer cases as part of the standard protocol, regardless of CA-125 levels or tumor volume. Some prior studies included only selected cases for lymph node dissection, and the rationale for selection was sometimes not clearly specified. Although our study did not include para-aortic lymph node dissection, the frequency of isolated para-aortic metastasis in low-risk cases is generally reported to be low [20]. Therefore, pelvic lymph node dissection alone is likely sufficient to detect lymph node metastasis in this cohort.

The main limitations of this study are its single-institution, retrospective design and relatively small number of cases. However, considering the rising worldwide adoption of sentinel lymph node navigation surgery, uniform systematic lymph node dissection for all low-risk patients is expected to become less common. Thus, even a single-institution retrospective study like ours, where all low-risk cases underwent pelvic lymph node dissection, retains its significance. Among the initially low-risk cases, 26.5% were upstaged after postoperative pathological diagnosis; nevertheless, the recurrence rate was only 2.6% (three cases) with a median follow-up of 40 months. The overall rate of lymph node metastasis (5.9%) was also in line with earlier studies [1].

## Conclusions

In low-risk endometrial cancer, defined as no more than half myometrial invasion, endometrioid carcinoma G1 or G2, and no extrauterine spread on preoperative assessment, a tumor volume index ≥38 cm^3^ emerged as a significant predictive factor for pelvic lymph node metastasis. These findings may aid clinical decision-making regarding whether to perform pelvic lymph node dissection and are also relevant for preoperative risk assessment in sentinel lymph node navigation surgery for low-risk endometrial cancer.
